# Estimating Energy Dissipation Rate from Breaking Waves Using Polarimetric SAR Images

**DOI:** 10.3390/s20226540

**Published:** 2020-11-16

**Authors:** Rafael D. Viana, João A. Lorenzzetti, Jonas T. Carvalho, Ferdinando Nunziata

**Affiliations:** 1 Earth Observation and Geoinformatics Division, National Institute for Space Research (OTG/INPE), São José dos Campos 12201-970, SP, Brazil; rafael.viana@inpe.br; 2 Laboratory of Ocean and Atmosphere Studies (LOA), Earth Observation and Geoinformatics Division, National Institute for Space Research (OTG/INPE), São José dos Campos 12201-970, SP, Brazil; jonas.carvalho@inpe.br; 3 Dipartimento di Ingegneria, Universitá degli Studi di Napoli Parthenope, 80143 Napoli, Italy; ferdinando.nunziata@uniparthenope.it

**Keywords:** SAR, radar polarimetry, wave breaking, energy dissipation rate

## Abstract

The total energy dissipation rate on the ocean surface, $\epsilon_{t}$ (W m${}^{-2}$), provides a first-order estimation of the kinetic energy input rate at the ocean–atmosphere interface. Studies on the spatial and temporal distribution of the energy dissipation rate are important for the improvement of climate and wave models. Traditional oceanographic research normally uses remote measurements (airborne and platforms sensors) and in situ data acquisition to estimate $\epsilon_{t}$; however, those methods cover small areas over time and are difficult to reproduce especially in the open oceans. Satellite remote sensing has proven the potential to estimate some parameters related to breaking waves on a synoptic scale, including the energy dissipation rate. In this paper, we use polarimetric Synthetic Aperture Radar (SAR) data to estimate $\epsilon_{t}$ under different wind and sea conditions. The used methodology consisted of decomposing the backscatter SAR return in terms of two contributions: a polarized contribution, associated with the fast response of the local wind (Bragg backscattering), and a non-polarized (NP) contribution, associated with wave breaking (Non-Bragg backscattering). Wind and wave parameters were estimated from the NP contribution and used to calculate $\epsilon_{t}$ from a parametric model dependent of these parameters. The results were analyzed using wave model outputs (WAVEWATCH III) and previous measurements documented in the literature. For the prevailing wind seas conditions, the $\epsilon_{t}$ estimated from pol-SAR data showed good agreement with dissipation associated with breaking waves when compared to numerical simulations. Under prevailing swell conditions, the total energy dissipation rate was higher than expected. The methodology adopted proved to be satisfactory to estimate the total energy dissipation rate for light to moderate wind conditions (winds below 10 m s${}^{-1}$), an environmental condition for which the current SAR polarimetric methods do not estimate $\epsilon_{t}$ properly.

## 1. Introduction

Wave breaking plays a significant role in momentum, energy and gas exchanges at the ocean–atmosphere interface. Wave breaking controls the maximum height of surface waves, and therefore can affect the skill of operational wave models [1]. The flux of greenhouse gases have been shown to depend on the water-side turbulence [2,3], which depends on the wave breaking [4,5,6]. The breaking of surface waves also produces whitecaps, promotes the mixing and enhances the insertion of bubbles in the surface layers in the ocean [1]. This influences the estimation of the inherent optical properties of the ocean, and thus the interpretation of the ocean color data [7,8]. Moreover, whitecap cover serves as a first step in the modeling of sea spray droplet as a sea spray source [9]. Several authors have demonstrated that instead of the wind speed, $U_{10}$, the total energy dissipation rate, $\epsilon_{t}$, is a more adequate parameter to model the whitecap coverage, since whitecaps are generated by the breaking of the waves [10,11,12].

Direct measurements of the energy dissipation rate resulting from wave breaking are not yet possible. However, estimates of $\epsilon_{t}$ can be obtained using spectral dissipation models. Different spectral dissipation models can be found in the literature [13,14,15,16]. Phillips [14] obtained the total energy dissipation rate by integrating the spectral density function over an equilibrium range of wavenumbers in which the wind input, dissipation and non-linear transfers were in local balance. This solution has been used as a basis for calculating the dissipation rate associated with breaking waves using field measurements of the wave spectrum [12,17,18,19]. In fact, the Phillips model has been used in measurements of the energy dissipation rate using remote sensing methods [20]. Several approaches using remote sensing can be used to study wave breaking properties, including: combined use of visual observations and simultaneous measurements of wind and waves [8,18,21], acoustic methods based on the penetration of air bubbles during the process of breaks [17,22], infrared remote sensing [23,24] and of the passive and active microwaves [9,25,26]. The difficulty in obtaining in situ measurements of wave breaking events and surface sea roughness, especially in strong wind fields, encourages the use of orbital remote sensing to derive an estimate of these quantities.

Polarimetric SAR data can be used to estimate the effects of breaking waves in co-polarized and cross-polarized radar return [27,28,29]. The main parameter of interest estimated by SAR is the oceanic normalized radar cross section (NRCS), also known as sigma zero ($\sigma_{0}$), which is proportional to the surface roughness on the scale of short waves. The $\sigma_{0}$ can be decomposed as a sum of polarized scattering, due to two-scale resonant Bragg-scattering, and a non-polarized scattering component (NP), due to breaking of waves [28,29]. Using RadarSAT-2 dual and quad-polarization, Hwang et al. [28] obtained an empirical relationship between the NP backscatter from cross-polarization return and the total energy dissipation rate. The authors found that quad-pol data is more accurate in estimating $\epsilon_{t}$, especially in conditions of strong winds ($U_{10}$ above 10–15 m s${}^{-1}$), where its low noise floor (about −36 dB) has a negligible contribution to the cross-polarized return. However, estimates of $\epsilon_{t}$ in light to moderate wind speed conditions using both dual-pol and quad-pol data does not have a good correlation with expected values from parametric models. Considering that whitecaps occur on the surface of the ocean under wind speeds above 3 m s${}^{-1}$ [18,30], the relationship obtained by Hwang et al. [28] does not include the interval between low-to-moderate wind speeds.

For low winds below 10 m s${}^{-1}$, Kudryavtsev et al. [31] observed that the NP contribution in co-polarized backscattering channels depends exponentially on the wind speed. More recently, Kudryavtsev et al. [32] derived empirical dependencies between the NP contribution in dual co- and cross-polarized SAR and wind speed and geometry of SAR observations (polarimetry, incidence and azimuth angles) using a large dataset of RadarSAT-2 quad-polarized images. The empirical functions obtained by the authors proved to be valid for wind speeds above 3 m s${}^{-1}$, and can then be used to assess upper ocean processes. Using this approach, this study aims to analyze the potential use of NP contribution presented in the co-polarized backscattering SAR return to estimate the total energy dissipation rate, extending the interval of estimation for the low-to-moderate winds. For this purpose, we use quad-pol RadarSAT-2 satellite images to estimate the total energy dissipation rate associated with breaking waves from the NP contribution present in the co-polarized NRCS channels. We analyze the estimated $\epsilon_{t}$ in relation to the Bragg polarization ratio model, and the influence of different environmental conditions and incidence angles. An indirect check of the proposed methodology was done comparing the estimates from SAR scenes with those obtained from wave modeling.

This paper is organized as follows. The study area and data are presented in Section 2. In Section 3 we present the methodology for estimating the total energy dissipation rate using backscatter SAR return. The results based on the proposed methodology are presented in Section 4, followed by the discussion in Section 5. The conclusions are presented in Section 6.

## 2. Study Area and Materials

### 2.1. Study Area

**Figure 1:**
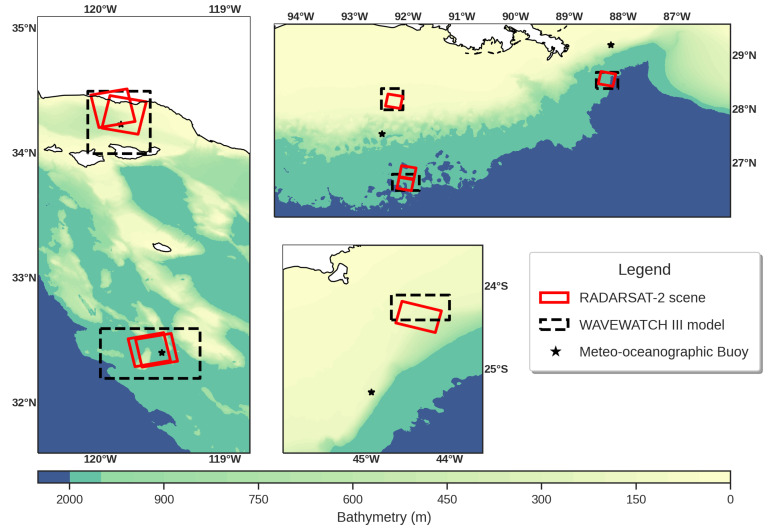
Study area used: (**left**) California coast; (**right and upper**) northern portion of the Gulf of Mexico; and (**right and bottom**) portion of the Santos Basin, Brazil.

This study was undertaken in three different oceanic regions: (1) a region off the California coast located in the South of the Santa Barbara Channel, (2) in the northern portion of the Gulf of Mexico, and (3) a region off the Santos Basin located close to the shelf break, Figure 1. These regions were chosen for their different marine and meteorological conditions, and proper for assessing the validity of the proposed methodology. The scenes were also obtained for different ocean depths, ranging from 20 to 1000 m.

### 2.2. SAR Data Set

The SAR data set consists of nine RadarSAT-2 (RS-2) fine-quad polarimetric acquisitions collected over different oceanic regions of the Atlantic and Pacific Ocean (see Figure 1, represented by red squares). Angles of incidence (AoI) ranging from about 24.6° in California coast to 43.3° in the northern part of the Gulf of Mexico. The RS-2 operates in the C-band (central frequency of 5.40 GHz) and has a nominal resolution of 4.7 and 5.2 m in the range and azimuth, respectively. Each scene covers an area of approximately 25 × 25 km, and the single-look complex (SLC) product provides the four complex scattering amplitudes, i.e., both co-pol channels (VV/HH) and cross-pol channels (VH/HV). The nominal noise-equivalent sigma zero (NESZ) of the system is approximately −36 dB but shows a variation with incidence angle [33].

**Figure 2:**
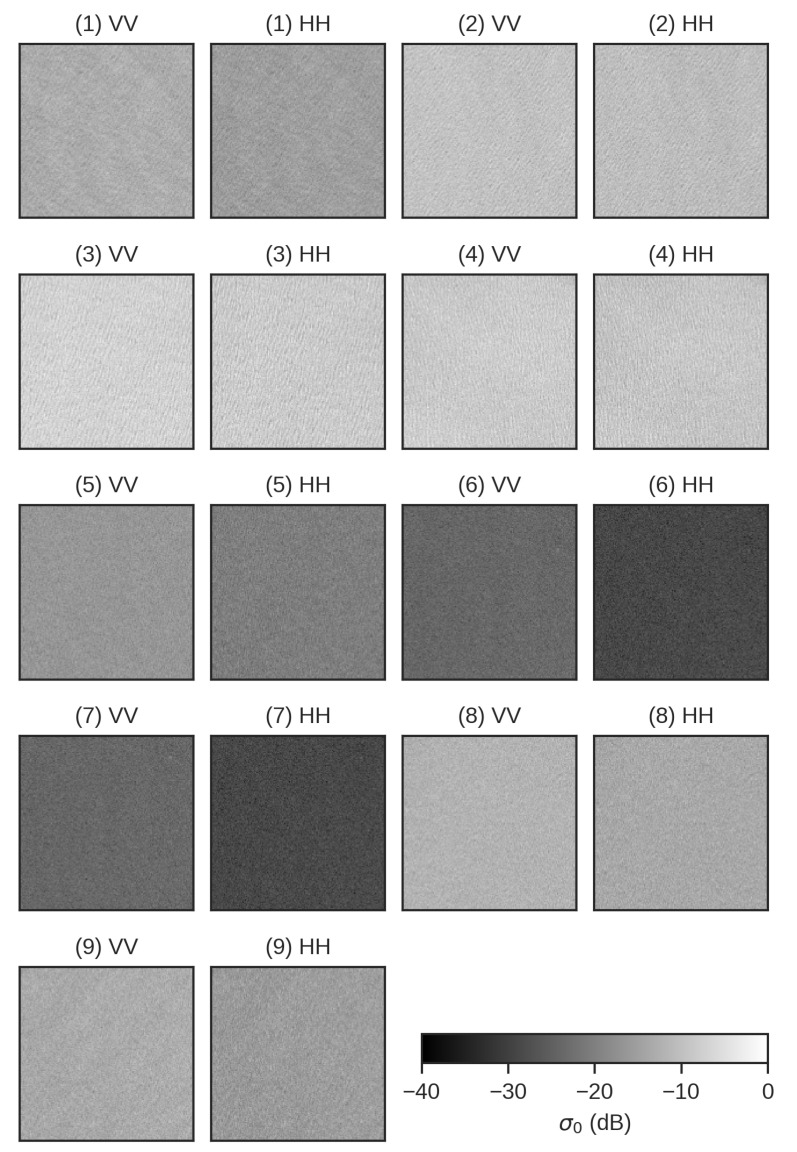
RadarSAT-2 dataset. VV- and HH-polarized intensity images (size: 150 × 150 pixels, spatial resolution: 50 m), in dB, over clean-sea surface area. Numbers refer to ID of each scene as shown in Table 1.

The original SAR data were pre-processed according to the following steps: (a) calibration in normalized radar cross section (NRCS) units, (b) NESZ correction using the method of the minimum eigenvalue of the 4 × 4 Coherency Matrix proposed in Hajnsek et al. [34], (c) extraction of clean-sea surface tiles from the SAR data set, and (d) finally the resampling of SAR images using the spatial *multilooking* with 10-by-10 pixels window. Figure 2 shows extracted tiles of the VV- and HH-polarized intensity images of SAR dataset representing clean-sea surface areas.

### 2.3. Ancillary Information

Wind field ($U_{10}$, $U_{dir}$) and sea surface temperature (SST) information was provided by the European Center for Medium-Range Weather Forecasts (ECMWF) ERA5 Reanalysis [35]. The wind and SST products from ERA5 data set are on a 0.25° × 0.25° spatial grid and hourly temporal resolution. The wind speed ranged from low-to-moderate wind speeds, i.e., from about 3 to 11 m s${}^{-1}$. Hereafter we use $U_{10}$ to refer to the low-resolution (∼25 km) wind speed field obtained from ERA5.

In situ wind and wave information was provided by the buoy data set from National Data Buoy Center (NDBC) and National Buoy Program of the Brazilian Navy (PNBOIA). This information was used to characterize the sea state for each scene, using the concept of a non-dimensional wave age [36], $\mathrm{WA}=c_{p}/u_{*}$, where $c_{p}$ is peak wave speed and $u_{*}$ is the friction velocity. Only half of the SAR scenes were collocated with the buoys, the other half of buoys were within 100 km from the scene. The used ancillary information is summarized in Table 1.

### 2.4. Wave Model

For this study we employed the third-generation wave model, WAVEWATCH III (WW3) version 5.16 [37], and used the model generated energy dissipation rate to compare to the satellite estimates. The WW3 model was developed by the National Oceanic and Atmospheric Administration/National Centers for Environmental Prediction (NOAA/NCEP) and it is currently at version 6.07.1. The forcing fields are 0.25° spatial resolution from ERA5 and the bottom topography data are from ETOPO1. The model was run using three nested grids in a two-way nesting scheme: a global grid of 25 km spatial resolution, a regional grid of 5 km spatial resolution, and a local grid of 1 km spatial resolution incorporating all or part of each SAR image (see Figure 1, represented by black rectangles).

The ST4 parameterization of Ardhuin et al. [16] for the input ($S_{in}$) and dissipation ($S_{ds}$) source term was used, while the non-linear interaction source term used was the Discrete Interaction Approximation (DIA) parameterization. The ST4 parameterization is based on [15], where dissipation source is due to breaking while dissipation by swell is considered a negative wind input. The dissipation source term is then calculated as the sum of two contributions: one breaking-induced contribution that is based on the local saturation spectrum and one cumulative dissipation rate contribution estimated from breaking wave probabilities. The full physical-mathematical description of this parameterization can be found in [16].

The model was configured to generate source term outputs every full hour using approximately 100 stations, distributed within each local grid and spaced between 1 to 2 km apart. The spectral grid was discretized with 24 directions and 25 frequencies.

## 3. Model Approach

### 3.1. Energy Dissipation Rate

Wave breaking is considered to be the dominant source of turbulence and energy dissipation in the upper ocean, and different studies of the energy dissipation rate indicate a cubic wind speed dependence [12,14,17,18]. Hwang and Sletten [12] establish a parameterized dissipation function expressed in terms of wind speed, significant wave height and peak wave frequency:

(1) $$\begin{array}{c} \epsilon_{t}=\alpha \rho_{a}U_{10}^{3} \end{array}$$

where $\rho_{a}$ is the air density (=1.20 kg m${}^{-3}$, for air at 20 °C) and α is a parameter dependent on the sea state development. As the wave develops, α gradually increases until it reaches a maximum value (∼5.7×10${}^{-4}$) in conditions of fully developed sea, and then decreases as the waves become more mature. The value of α depends on the non-dimensional parameters of peak wave frequency and surface elevation variance. For convenience, these parameters can be approximated from $U_{10}$ using the look-up-table described in Hwang and Sletten [12] (see their Table 1).

Wind speed, surface elevation variance and peak wave frequency can be obtained using satellite remote sensing (e.g., scatterometry and altimetry) or meteo-oceanographic buoy data. However, such methods provide only the regional wind and wave fields due to their low spatial resolution. The high spatial resolution from SAR data has the potential to estimate the energy dissipation rate on a local scale.

### 3.2. Wave Breaking Contribution in $\sigma_{0}$

Using the concept of length of breaking fronts per unit area statistics introduced by [38] combined with an approach of the composite Bragg theory, Kudryavtsev et al. [27,29] described a model to predict $\sigma_{0}$ as a combination of three components:

(2) $$\begin{array}{ccc} \sigma_{0}^{pp} & = & \sigma_{0Br}^{pp}+\sigma_{sp}^{pp}+\sigma_{wb} \end{array}$$

where *pp* represents the transmitted and received polarizations (H or V). The first component on right side, $\sigma_{0Br}^{pp}$, represents backscatter associated with the Bragg’s resonant mechanism. The second component, $\sigma_{sp}^{pp}$, is associated with the contribution of specular reflection, which can be neglected for intermediate incidence angles. The last component, $\sigma_{wb}$, represents the non-polarized (NP) contribution due to the breaking of surface waves, and it is the same for both polarizations [27]. The wave breaking contribution can be removed through the polarization difference (PD) between co-polarized returns:

(3) $$\begin{array}{ccc} \Delta \sigma_{0} & = & \sigma_{0}^{VV}-\sigma_{0}^{HH}\;=\;\sigma_{0Br}^{VV}-\sigma_{0Br}^{HH} \end{array}$$

Combining Equations (2) and (3), and considering the range of incidence angles between 25° and 50° where the specular contribution can be neglected, the NP contribution can be expressed as a function of $\Delta \sigma_{0}$ as:

(4) $$\begin{array}{ccc} \sigma_{wb} & = & \sigma_{0}^{VV}-\frac{\Delta \sigma_{0}}{1-p_{B}} \end{array}$$

where $p_{B}$ correspond to the polarization ratio for the two-scale Bragg-scattering components ($p_{B}=\sigma_{0Br}^{HH}/\sigma_{0Br}^{VV}$). Using Equations (3) and (4), the co-polarized images can be decomposed in NP and PD components which are related to different scattering mechanisms. The NP contribution is related to enhanced surface roughness generated by the breaking waves and also specular reflection from forward faces of breaking waves, while the PD contribution is related to Bragg scattering provided by wind waves responses [31,32]. The PD contribution is estimated directly from the $\sigma_{0}$ measurements in each SAR image. On the other hand, the Bragg polarization ratio cannot be estimated directly from the scenes. Different approaches can be used to obtain $p_{B}$. Two possibilities to evaluate $p_{B}$ are:

(a) using simplified two-scale models (TSM) calculated from the mean square slope (MSS) statistics of tilting waves [27,29]; or,
(b) estimated using $\sigma_{0Br}^{VV}$ and $\sigma_{0Br}^{HH}$ from an electromagnetic backscatter model calculated from a chosen wave spectrum model [39,40].

Although Method (a) using approximate equations is computationally simpler, Method (b) should provide a more accurate estimate of $p_{B}$ at the cost of more computational time depending on the electromagnetic model used [41]. In our work, $\sigma_{wb}$ was calculated from SAR images using Equation (4), pixel-at-pixel, with the Bragg polarization ratio estimated using both methods (a) and (b). We used the approximate equations for the TSM model described in Kudryavtsev et al. [32] (see Equation (A1) in Appendix A) for Method (a), and we used the two-scale Boundary Perturbation Model (BPM) [42,43] for Method (b).

The BPM model represents the backscattering ($\sigma_{0}$) of the sea surface as a superposition of two independent stochastic processes: one process associated with capillary and short–gravity waves (small-scale roughness) and one process associated with the long waves (large-scale roughness). It has been used for sea surface scattering [42] and sea oil slick observations [43,44], and the model allows for detailed scattering calculations in very little computer time in a table-top machine. The full physical-mathematical description of BPM model can be found in [43]. The two-dimensional sea surface spectrum used for the BPM model was Elfouhaily [45], which is specified once the wind friction velocity (calculated from $U_{10}$ and formula described in [46]) and the fetch *x* are provided.

### 3.3. Relationship between $\sigma_{wb}$ and Environmental and Imaging Parameters

Using nearly 1700 RS-2 quad-pol SAR images co-located with buoy observations and the model described by Equation (4), Kudryavtsev et al. [32] derived an empirical relationship between $\sigma_{wb}$ and wind speed $U_{10}$, incidence angle θ and azimuth angle ϕ, as (see their Equation (8)):

(5) $$\begin{array}{c} \sigma_{wb}=f_{wb}\left(\theta \right)Y_{wb}\left(\phi \right)U_{10}^{n_{wb}\left(\theta \right)} \end{array}$$

where $f_{wb}$ and $n_{wb}$ depends on incidence angle, and are described as:

(6) $$\begin{array}{ccc} f_{wb}\left(\theta \right) & = & 1.9\times 10^{-3}e^{-0.32(\theta -30^{{}^{\circ}})}\\ n_{wb}\left(\theta \right) & = & 1.3+4.7\times 10^{-2}\left(\theta -30^{{}^{\circ}}\right) \end{array}$$

The angular distribution coefficient, $Y_{wb}$, depends on both θ and ϕ, and is described as:

(7) $$\begin{array}{c} Y_{wb}\left(\phi \right)=\exp\left[A_{0wb}+A_{1wb}\cos\phi +A_{2wb}\cos2\phi \right]\\ \mathrm{with},\;\left\{\begin{array}{c} A_{0\mathrm{wb}}=0.24-1.4\times 10^{-2}\left(\theta -30^{{}^{\circ}}\right)\\ A_{1\mathrm{wb}}=0.33+1.3\times 10^{-2}\left(\theta -30^{{}^{\circ}}\right)\\ A_{2\mathrm{wb}}=0.12+1.4\times 10^{-2}\left(\theta -30^{{}^{\circ}}\right) \end{array}\right. \end{array}$$

Based on these results, $\sigma_{wb}$ can be estimated directly from the SAR co-polarized components (Equation (4)) or from the environmental and imaging conditions (Equation (5)). We use the NP contribution obtained from Equation (4) at each pixel of the SAR scenes to estimate the associated wind speed (${\tilde{U}}_{10}$) using Equations (5)–(7) as:

(8) $$\begin{array}{c} {\tilde{U}}_{10}\left(\sigma_{wb},\theta ,\phi \right)={\left(\frac{\sigma_{wb}}{f_{wb}\left(\theta \right)Y_{wb}\left(\phi \right)}\right)}^{1/n_{wb}\left(\theta \right)} \end{array}$$

In this way, we preserve the variability associated with wind speed while taking into account the effect of wave breaking. Hereafter we use ${\tilde{U}}_{10}$ to refer to the high-resolution (∼500 m) wind speed field obtained from SAR images and Equation (8). Finally, we used ${\tilde{U}}_{10}$, instead of $U_{10}$ from ERA5 data set, to estimate the α parameter, now function of ${\tilde{U}}_{10}$, and the total energy dissipation rate from Equation (1), as:

(9) $$\begin{array}{c} \epsilon_{t}=\alpha \left({\tilde{U}}_{10}\right)\rho_{a}{\tilde{U}}_{10}^{3} \end{array}$$

thus obtaining the energy dissipation rate at ∼500 m of spatial resolution.

## 4. Results

### 4.1. Influence of the $p_{B}$ Estimation on the Non-Polarized Contribution

**Figure 3:**
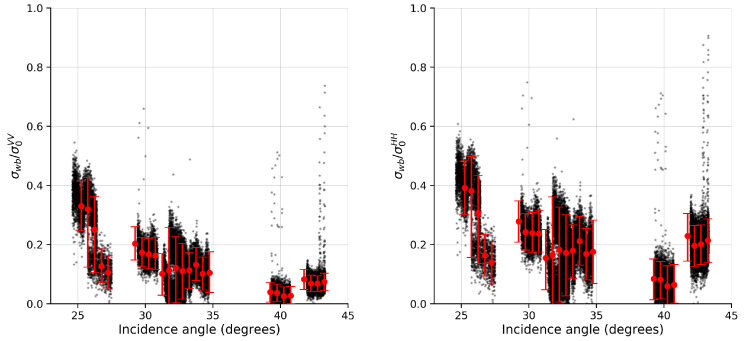
Relative contribution of $\sigma_{wb}$ in VV (**left**) and HH (**right**) components when using a simplified TSM model. RadarSAT-2 observations are represented as black dots, while red circles with vertical bars are mean values and percentiles of 5% and 95%, respectively.

**Figure 4:**
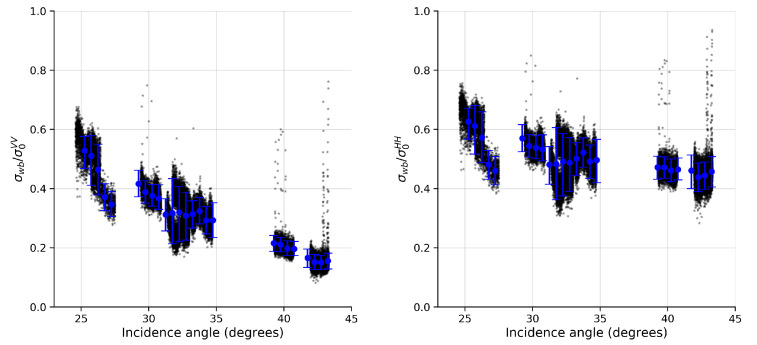
Relative contribution of $\sigma_{wb}$ in VV (**left**) and HH (**right**) components when using a BPM model. RadarSAT-2 observations are represented as black dots, while blue circles with vertical bars are mean values and percentiles of 5% and 95%, respectively.

The relative contribution of $\sigma_{wb}$ in the co-polarized components as a function of the incidence angle, when using (a) the simplified TSM model (Appendix A) and (b) BPM model to estimate the polarization ratio, is shown in Figure 3 and Figure 4, respectively. Using the simplified TSM model (Figure 3), the relative contribution for both polarizations varied between 10% and 50%, with the lowest values occurring mostly in the high incidence angles. The percentual values obtained in our work are below the average values obtained by [32]. Using the BPM model (Figure 4), the relative contribution in VV varied between 20% and 70%, with the highest percentage of contribution occurring at low incidence angles (approximately 60%), and rapidly decreasing to approximately 15% at high incidence angles. The relative contribution in HH, however, remained at approximately 50% across the range of moderate incidence angles (>30°), with the maximum contribution occurring at low incidence angles. These results show a similar behavior to those obtained by [32], where the contribution of $\sigma_{wb}$ in VV-polarization shows a decreasing trend with increasing incidence angle, and the contribution in HH-polarization tends to stabilize at incidence angles above 30°. In addition, the percentage values are closer to the average values obtained by the authors (see [32], Figure 2) when using the BPM model.

**Figure 5:**
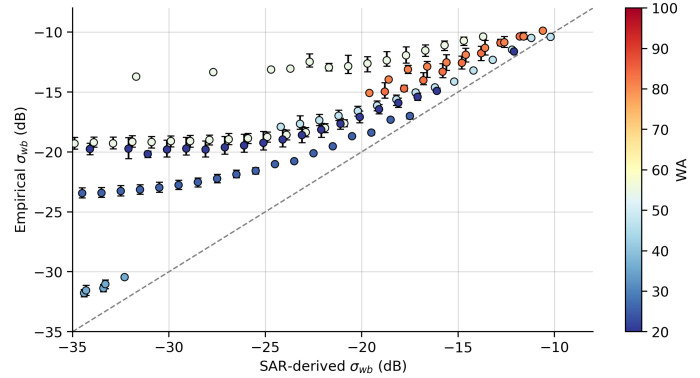
Comparison between $\sigma_{wb}$ estimated using Equation (5) (Empirical $\sigma_{wb}$) versus $\sigma_{wb}$ estimated from SAR images (SAR-derived $\sigma_{wb}$) using a simplified TSM model. The observations were grouped into 1 dB bins, where the circles with vertical bars are mean values and percentiles of 5% and 95%. Colors represent the wave age of observations (see Table 1).

**Figure 6:**
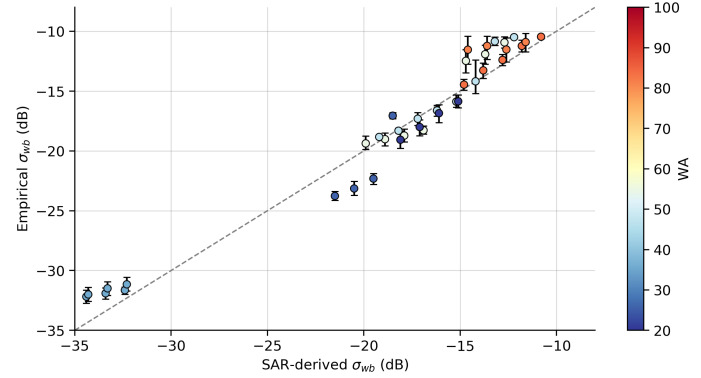
Comparison between $\sigma_{wb}$ estimated using Equation (5) (Empirical $\sigma_{wb}$) versus $\sigma_{wb}$ estimated from SAR images (SAR-derived $\sigma_{wb}$) using the BPM model. The observations were grouped into 1 dB bins, where the circles with vertical bars are mean values and percentiles of 5% and 95%. Colors represent the wave age of observations (see Table 1).

The sensitivity of $\sigma_{wb}$ estimated from SAR images (SAR-derived $\sigma_{wb}$) with respect to the method used to determine $p_{B}$ was analyzed using the expected value of $\sigma_{wb}$ using the environment low-resolution wind ($U_{10}$, $U_{dir}$) and imaging (AoI, azimuthal angles) configurations and the empirical relationship described in Equation (5) (Empirical $\sigma_{wb}$). Figure 5 and Figure 6 show a comparison of $\sigma_{wb}$ estimated using both the simplified TSM and BPM models. As already noted in Figure 3, there was an underestimation of the NP contribution when using the TSM model, in all sea conditions. Kudryavtsev et al. [31] showed that a variation of up to ±10% in the estimate of $p_{B}$ can lead to an error of approximately ±20% or greater in the estimate of NP contribution. The estimate of the NP contribution when using the BPM model (Figure 6) showed an overall good agreement with the estimated values using the empirical relationship, even at and especially in sea conditions of prevailing old swell. The estimates obtained from the simplified TSM model had bias values of −4.45 dB and RMSE of 5.45 dB, indicating both an underestimation and a large variance in $\sigma_{wb}$ in relation to the expected average in each scene. The estimates obtained from the BPM model had values of bias of −0.33 dB and RMSE of 1.99 dB, which were close to the values obtained by Kudryavtsev et al. [32].

### 4.2. Estimation of $\epsilon_{t}$ Using the Non-Polarized Contribution

**Figure 7:**
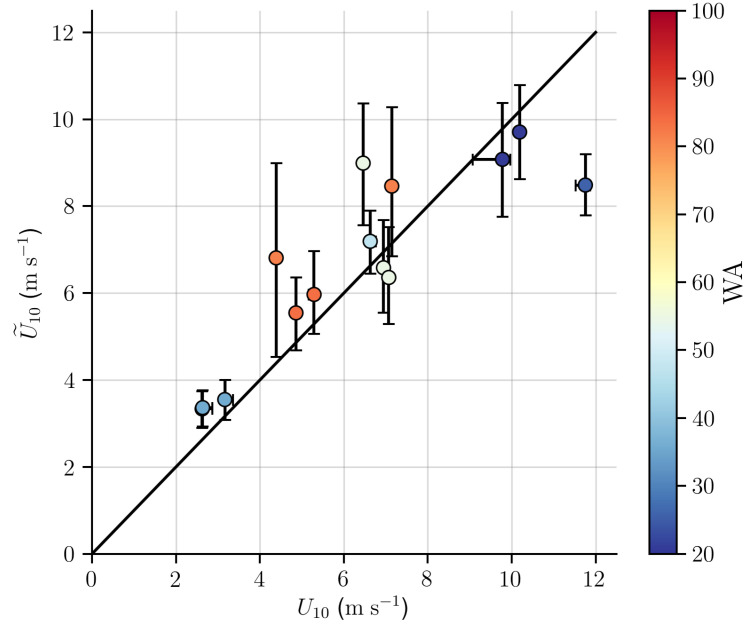
Comparison between ($U_{10}$) wind speed obtained from ERA5 and (${\tilde{U}}_{10}$) associated wind speed derived from the SAR images with the BPM model using Equation (8). The observations were grouped into 0.5 m s${}^{-1}$ bins, where the circles are mean values. Vertical and horizontal bars represent percentiles of 5% and 95%. Colors represent the wave age of observations (see Table 1).

A comparison between associated wind speed (${\tilde{U}}_{10}$) derived from the SAR images with the BPM model using Equation (8) and wind speed obtained from ERA5 ($U_{10}$) is presented in Figure 7. It can be observed a greater variability in ${\tilde{U}}_{10}$ values in relation to the same interval of $U_{10}$ values. Although on average ${\tilde{U}}_{10}$ correspond to the wind field expressed by $U_{10}$ on regional scale, the latter is not able to represent the local variability associated with wind stress on the ocean surface.

**Figure 8:**
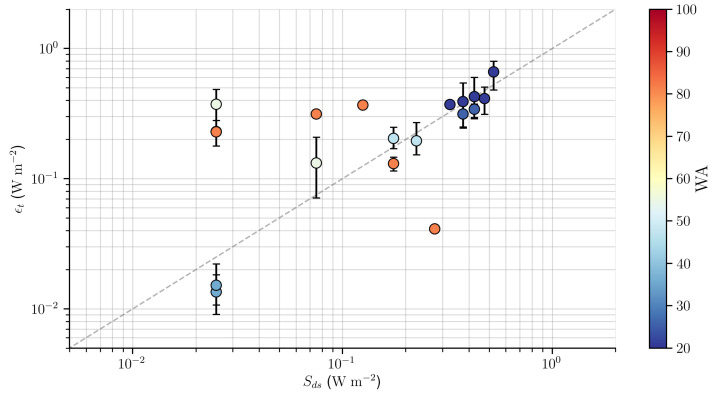
Comparison between total energy dissipation rate derived from SAR images ($\epsilon_{t}$) and wave dissipation rate calculated by WW3 model ($S_{ds}$). The observed $\sigma_{wb}$ were grouped into 1 dB bins, where the circles with vertical bars are mean values and percentiles of 5% and 95%. Colors represent the wave age of observations (see Table 1).

A comparison between $\epsilon_{t}$ derived from the SAR images with the BPM model and the wave dissipation rate $S_{ds}$ integrated over all wavenumbers resolved by WW3 model is presented in Figure 8. The observed $\sigma_{wb}$ were grouped into 1 dB bins, where circles with vertical bars are mean values and percentiles of 5% and 95% respectively. The wave age is indicated by the color scale, ranging from young wind-sea waves to old swell waves [47].

In wind-sea wave conditions (WA ≲ 40) the relationship between $\epsilon_{t}$ and $S_{ds}$ proved to be approximately linear. In swell-dominated conditions (WA ≳ 40), it is possible to observe that in general $\epsilon_{t}$ was much higher than $S_{ds}$ by almost an order of magnitude, and showed a much greater scattering around the 1:1 line.

**Figure 9:**
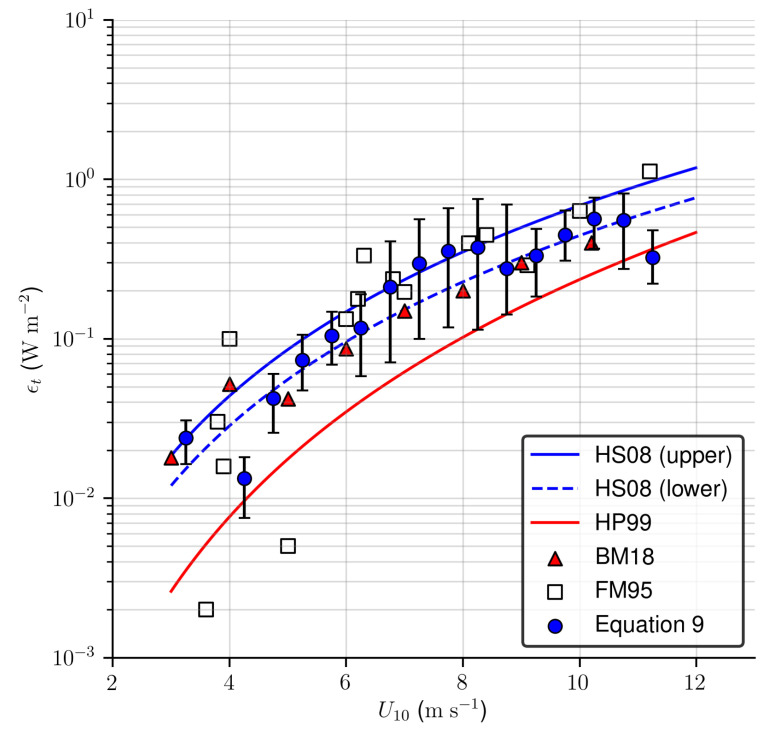
Relationship between total energy dissipation rate estimated from the SAR data versus $U_{10}$, averaged in 0.5 m s${}^{-1}$ wind speed bins. Blue circles with vertical bars are mean values and percentiles of 5% and 95%. Two parameterizations of $\epsilon_{t}$ as a function of wind speed are shown, taken from [18] (HP99, red curve) and [26] (HS08, blue curves). Field estimation measurements taken from [17] (FM95, white squares) and [19] (BM18, red triangles) were also included.

The relationship between $\epsilon_{t}$ and $U_{10}$ estimated from the SAR data is shown in Figure 9. For comparison, two parameterizations of the energy dissipation rate as a function of $U_{10}$ are shown, which are taken from Hanson and Phillips [18] (HP99, red curve) and Hwang and Sletten [12] (HS08, blue curves). The α parameter used in HS08 parameterization corresponds to young stages of wave development or swell-dominated seas (lower, α = 3.7) and at fully developed seas (upper, α = 5.7). Field estimation measurements reported by Felizardo and Melville [17] (FM95, white squares) and Banner and Morison [19] (BM18, red triangles) are also included.

Comparing the results obtained in this work with the field estimates, we can observe that there is a good agreement between $\epsilon_{t}$ values, especially when compared to the measurements of FM95 and BM18. The methodology used was also able to identify the variability associated with different sea states for the same wind speed range. At wind speeds below 10 m s${}^{-1}$, our estimates were below the maximum value expected in peak conditions of saturated seas (upper curve HS08), while the parametrization of HP99 proved to be a lower limit for the estimated values. It is important to note that the local wind history was not taken into account in the present study, and therefore the effects of wind time variation on the contribution of $\epsilon_{t}$ could not be assessed, as suggested by [18,48].

## 5. Discussion

### 5.1. Sensitivity of $\epsilon_{t}$ in Relation to $p_{B}$ Determination

Comparing the results here obtained with those obtained by [32,49], we observe that the percent contribution of $\sigma_{wb}$ in the VV-polarization shows a similar decreasing behavior in relation to the angle of incidence, while the contribution for HH-polarization tends to stabilize at incidence angles above 30°. Differently from mean values obtained by [32], simplified TSM model underestimated the NP contribution when compared to the BPM model. This difference might be due to the approximate model that does not take into account second order Bragg-scattering effects [27,31]. Several studies confirm that empirical models of the Bragg polarization ratio tend to overestimate $p_{B}$ when dependent only on the angle of incidence [49,50] (see [32] Figure 1a).

We can also consider the hypothesis that the lower values of $\epsilon_{t}$ obtained using the simplified TSM model correspond to the lower than average values observed in [32]. Since no information was presented by the authors regarding the marine and meteorological conditions associated with these values, this hypothesis cannot be confirmed. As noted by [51], at low incidence angles specular reflection can lead to an increase in the NP component. Moreover, Mouche et al. [39] pointed out that in low wind speed conditions ($U_{10}$ < 5 m s${}^{-1}$) discrepancies between measured and predicted values can be observed by the used different backscattering models due to a series of assumptions, and that in this case can dominate the modeled return caused by breaking waves.

Another possible source of error in the estimation of $\sigma_{wb}$ can be the influence of NESZ. A parameter widely used to measure the impact of noise on the measured return is the signal-to-noise ratio (SNR) [52,53,54]. Espeseth et al. [54] suggests that the SNR must exceed a certain value (of the order of 10 dB) before any polarimetric analysis be performed, as the decomposition of the backscattering return done in this study. The analysis of NESZ is therefore necessary to guarantee that the NP contribution in co-polarized components is not affected by noise. For this analysis, regions of interests (ROIs) were extracted from each SAR scene and the same number of pixels (=900) were selected at random within from each ROI. The SNR was then calculated using the mean value of $\sigma_{0}$ and the mean value of NESZ of the pixels extracted from each ROI, as SNR = ($\sigma_{0}$− NESZ)/NESZ.

**Figure 10:**
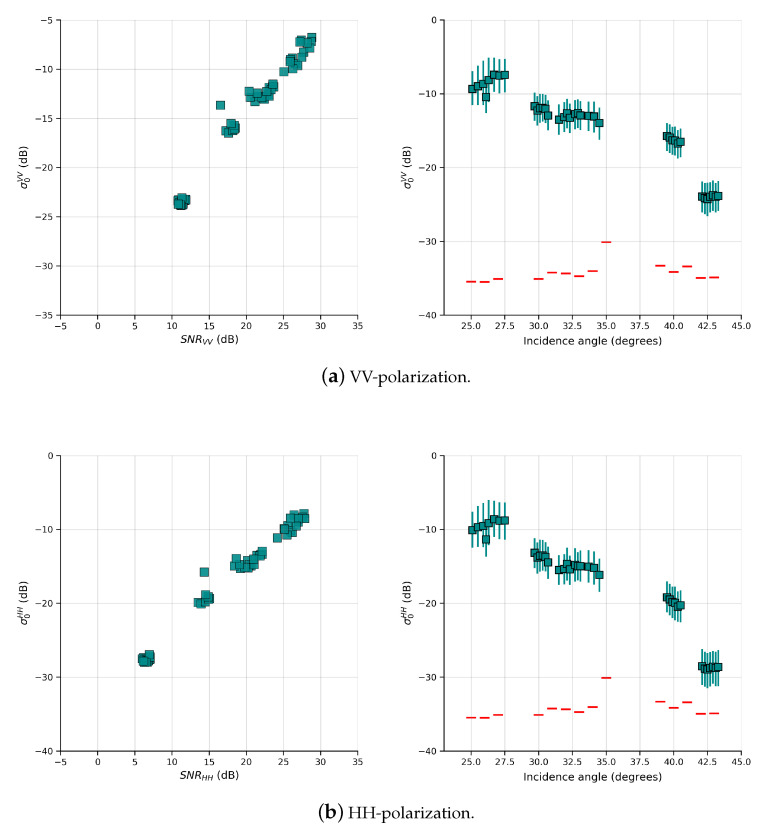
(**Left**) Mean $\sigma_{0}$ values versus the SNR in dB for VV (**a**) and HH (**b**) polarizations. (**Right**) Mean $\sigma_{0}$ values and percentiles of 5% and 95% versus the mean incidence angle in each ROI for VV (**a**) and HH (**b**) polarizations. Red lines represent the estimated NESZ as function of mean incidence angle.

Figure 10 shows the variation of the mean value of the $\sigma_{0}$ for HH and VV polarizations as a function of the SNR (left) and mean incidence angle (right). It is possible to observe that the values of SNR in VV-polarization were above 10 dB in all ROIs, and that none of the ROIs had $\sigma_{0}$ values below the NESZ (SNR equal to 0 dB), the smallest difference corresponding to approximately 10 dB at high incident angles (>42°). Regarding the HH-polarization, most ROIs have SNR above 10 dB while a few fall below this value. These ROIs are within scenes for which wind speed conditions had magnitude of 3 m s${}^{-1}$ at high angles of incidence, where backscatter returns are expected to be low and the noise is higher [54]. However, the measured signal remained 6 dB above of noise floor, which can be considered an adequate value and under minimum influence of noise [53]. Therefore, we can conclude that the differences in $\sigma_{wb}$ are not caused by noise and should be exclusively due to the methodology used to determinate $p_{B}$.

### 5.2. Comparison of $\epsilon_{t}$ with Wave Model Outputs

Two main factors may suggest the discrepancy of $\epsilon_{t}$ estimates on different wave conditions: (1) wave breaking may not be the dominant mechanism for wave dissipation, or (2) there was an overestimation of the $\sigma_{wb}$, and consequently of the $\epsilon_{t}$ for old-sea waves. Another possible source of uncertainty in the $\epsilon_{t}$ estimation comes from the estimation of α parameter, given the large uncertainty associate with this quantity that could in some cases be on the order of 100% [12].

Banner and Morrison [19] obtained similar results in the comparison between the contribution of the breaking wave dissipation rate to the total energy dissipation rate (Figure 8), when reanalyzing Sutherland and Melville [24] data set to investigate the relative contribution of microscale breakers to $\epsilon_{t}$. The authors found that wave breaking is responsible for almost all energy dissipation during the development of waves, and that in old seas the breaking of waves contributes only to a small fraction of $\epsilon_{t}$. The greatest contribution to the total energy dissipation rate would then be related mainly to other hydrodynamic processes, which include the influence of surface waves on the Reynolds shear, turbulence and the transfer of energy between waves at different scales [19].

There are not many studies that estimate the impact of the presence of swell waves on the value of $\sigma_{0}$ measured in different polarizations. Durden and Vesecky [55] evaluated the impact of the presence of swell in the HH and VV polarizations, at different SAR frequencies, and concluded that the effects are more significant at low frequencies (L band) and low incidence angles. At high frequencies (for example, K${}_{u}$ band) and high incidence angles, the effects were almost negligible. Hwang and Plant [40] analyzed the effect of waves at low-to-moderate incidence angles for different SAR frequencies, using their empirical wave spectrum, and found that the effect of swell on $\sigma_{0}$ is less than 3 dB for wind speeds above 4 m s${}^{-1}$, for HH and VV polarizations (C-band).

Results presented in [56] showed a strong correlation between the Bragg polarization ratio and wave steepness and significant wave height. When the steepness increases, the polarization ratio also increases. Still according to [56], the polarization difference has a very low correlation with the wave parameters. The $p_{B}$ estimates in [40] (see their Figure 5) also indicate an increase in the polarization ratio in the presence of swell. The main effect of this increase in the polarization ratio, however, corresponds to a greater variance in the NRCS measurements, as can be seen in the distribution of the values of the NP contribution in Figure 5. The combined results then indicate that swell is mainly responsible for the greatest variance in our estimates of $\epsilon_{t}$, and that the discrepancy between the values of $\epsilon_{t}$ and $S_{ds}$ are expected since in these cases the contribution to total energy dissipation comes from mechanisms other than the wave breaking.

## 6. Conclusions

In this study, we investigated the use of the SAR co-polarized data to estimate the energy dissipation rate associated with wave breaking processes in the ocean surface, particularly for the low to moderate wind conditions ($U_{10}$ below 10 m s${}^{-1}$). The dissipation source term estimated using numerical wave model (WW3) was adopted as a reference for energy dissipation due to the breaking of waves, while the SAR data set considers different imaging configurations and environmental conditions. Under prevailing swell conditions, there was a greater variance in the estimate of the NP contribution, and consequently in the rate of energy dissipation. This results can mean that: (i) previous results have found a low dissipation rate during swell, or (ii) the method overestimates the dissipation rate during swell, and these variations may be associated with the variation in the backscatter return due to the presence of the swell. However, this latter process was not quantified in the present work. We obtained satisfactory results in all sea conditions when compared with previous measurements reported in the literature, although in swell waves conditions was not possible to associate energy dissipation only with wave breaking processes.

The estimation of the Bragg polarization ratio proved to be one of the most important steps in this approach. The choice of an electromagnetic model that does not calculate properly the co-polarized backscatter tends to underestimate the NP contribution, and consequently the total energy dissipation rate. The present published models can adequately represent the VV-polarization, but generally are not as good in the HH-polarization. Electromagnetic models capable of more accurate estimates may require more computational time to process each image. The model adopted in this work, which was run on a normal desktop computer, was able to process the entire methodology with a low processing time (less than 10 min per scene).

The estimates of $\epsilon_{t}$ here derived were of the same order of magnitude as previous measurements published in the literature derived from in situ methods, with the advantage of being able to cover large areas of the ocean surface. With the increase in the number of satellites carrying polarimetric SAR sensors, this methodology offers one another possibility for an extensive estimation of wave breaking and energy dissipation from space.

## Figures and Tables

**Table 1:** RadarSAT-2 data set and meteo-oceanographic variables.

Scene	Product		Central Location		AoI		$U_{10}$	$U_{\mathrm{dir}}$	
ID	ID	Date/Hour (UTM)	Latitude	Longitude	(°)	Orbit	(m s${}^{-1}$)	(°)	WA
1	53,617	26 September 2009 01:56	32.43° N	119.54° W	31.3–33.0	Ascending	8.0	123	54.8
2	57,307	27 October 2009 01:52	32.43° N	119.61° W	24.6–26.5	Ascending	5.6	127	83.6
3	63,140	14 December 2009 01:52	34.36° N	119.90° W	25.7–27.6	Ascending	8.4	108	54.7
4	63,215	14 December 2009 14:09	34.31° N	119.80° W	24.6–26.5	Descending	4.2	172	123.4
5	79,608	1 May 2010 12:05	28.14° N	92.27° W	39.3–40.7	Descending	10.0	330	25.9
6	80,536	8 May 2010 12:01	26.80° N	92.02° W	41.9–43.3	Descending	6.0	270	35.7
7	80,536	8 May 2010 12:01	26.63° N	92.05° W	41.9–43.3	Descending	6.0	270	35.7
8	81,514	15 May 2010 11:57	28.39° N	88.34° W	41.9–43.3	Descending	7.3	295	47.1
9	496,265	5 August 2016 08:25	24.36° S	44.37° W	31.7–34.7	Descending	12.5	258	20.9

## Abbreviations

The following abbreviations are used in this manuscript:

AOI	Angle of incidence
BPM	Boundary perturbation model
CSA	Canadian Space Agency
dB	Decibel
ECMWF	European Center for Medium-Range Weather Forecasts
HH	horizontal transmit-horizontal receive
MDA	MacDonald Dettwiler and Associates
MSS	Mean square slope
NESZ	Noise-equivalent sigma zero
NP	Non-polarized
NRCS	Normalized radar cross section
NCEP	National Centers for Environmental Prediction
NDBC	National Data Buoy Center
NOAA	National Oceanic and Atmospheric Administration
PD	Polarized difference
PNBOIA	National Buoy Program of the Brazilian Navy
RS-2	RadarSAT-2
ROI	Region of interest
SAR	Synthetic aperture radar
SLC	Single-look complex
SST	Sea surface temperature
TSM	Two-scale model
VV	Vertical transmit-vertical receive
WW3	WAVEWATCH III

## Appendix A

Kudryavtsev et al. [27] showed that at moderate incidence angles (θ > 25°) and for small MSS of the tilting waves (waves with wavelength longer than a few times the Bragg wavelength), the Bragg polarization ratio can be significantly simplified to:

(A1) $$\begin{array}{ccc} p_{B} & = & \frac{|G_{HH}{|}^{2}}{|G_{VV}{|}^{2}}\times \frac{1+g_{HH}s_{i}^{2}}{1+g_{VV}s_{i}^{2}} \end{array}$$

where $G_{pp}$ are scattering coefficients for co-polarized components, $s_{i}^{2}$ is the MSS of tilting waves in the incidence plane direction, and $g_{pp}$ are polarization coefficients accounting for the impact of the tilting waves in the second order. Scattering coefficients for the sea surface (in C-Band) can be calculated from:

(A2) $$\begin{array}{ccc} |G_{VV}{|}^{2} & = & \frac{\cos^{4}\theta \left(1+\sin^{2}\theta \right)}{{\left(\cos\theta +0.111\right)}^{4}} \end{array}$$

(A3) $$\begin{array}{ccc} |G_{HH}{|}^{2} & = & \frac{\cos^{4}\theta }{{\left(0.111\cos\theta +1\right)}^{4}} \end{array}$$

assuming that the dielectric constant of seawater is large, in particular equal 81 [57]. Polarization coefficients, evaluated from (A2) and (A3), are defined as:

(A4) $$\begin{array}{ccc} g_{VV} & = & \frac{\tan^{4}\theta }{2|G_{VV}{|}^{2}}\frac{\partial^{2}}{\partial \theta^{2}}\left(\frac{|G_{VV}{|}^{2}}{\tan^{4}\theta }\right) \end{array}$$

(A5) $$\begin{array}{ccc} g_{HH} & = & \frac{\tan^{4}\theta }{2|G_{HH}{|}^{2}}\frac{\partial^{2}}{\partial \theta^{2}}\left(\frac{|G_{HH}{|}^{2}}{\tan^{4}\theta }\right)+\frac{2}{\sin^{2}\theta }\left|\frac{G_{VV}}{G_{VV}}\right| \end{array}$$

Assuming that the slopes of the waves with small MSS are almost isotropic [32], and using the empirical formulation described in [58], we can calculate the MSS by:

(A6) $$\begin{array}{ccc} s_{i}^{2} & = & 2.25\times 10^{-3}\ln\left(\beta^{-2}k_{br}U_{10}^{2}/4g\right) \end{array}$$

where $U_{10}$ is the wind speed from ERA5 data set, *g* is the gravity acceleration, $k_{br}$ is the Bragg wavenumber, $\beta =U_{10}{\left(k_{p}/g\right)}^{1/2}$ is the inverse wave age, and $k_{p}=g/U_{10}^{2}$ is the spectral peak wavenumber [59].
